# Adhesion of *Helicobacter* Species to the Human Gastric Mucosa: A Deep Look Into Glycans Role

**DOI:** 10.3389/fmolb.2021.656439

**Published:** 2021-05-07

**Authors:** Rita Matos, Irina Amorim, Ana Magalhães, Freddy Haesebrouck, Fátima Gärtner, Celso A. Reis

**Affiliations:** ^1^ Instituto de Investigação e Inovação Em Saúde (i3S), Universidade do Porto, Porto, Portugal; ^2^ Instituto de Patologia e Imunologia Molecular da Universidade do Porto (IPATIMUP), Porto, Portugal; ^3^ Instituto de Ciências Biomédicas Abel Salazar da Universidade do Porto (ICBAS), Porto, Portugal; ^4^ Department of Pathology, Bacteriology and Avian Diseases, Faculty of Veterinary Medicine, Ghent University, Ghent, Belgium; ^5^ Faculdade de Medicina da Universidade do Porto (FMUP), Porto, Portugal

**Keywords:** adhesion, gastric mucosa, glycans, glycosylation, *helicobacter*

## Abstract

*Helicobacter* species infections may be associated with the development of gastric disorders, such as gastritis, peptic ulcers, intestinal metaplasia, dysplasia and gastric carcinoma. Binding of these bacteria to the gastric mucosa occurs through the recognition of specific glycan receptors expressed by the host epithelial cells. This review addresses the state of the art knowledge on these host glycan structures and the bacterial adhesins involved in *Helicobacter* spp. adhesion to gastric mucosa colonization. Glycans are expressed on every cell surface and they are crucial for several biological processes, including protein folding, cell signaling and recognition, and host-pathogen interactions. *Helicobacter pylori* is the most predominant gastric *Helicobacter* species in humans. The adhesion of this bacterium to glycan epitopes present on the gastric epithelial surface is a crucial step for a successful colonization. Major adhesins essential for colonization and infection are the blood-group antigen-binding adhesin (BabA) which mediates the interaction with fucosylated H-type 1 and Lewis B glycans, and the sialic acid-binding adhesin (SabA) which recognizes the sialyl-Lewis A and X glycan antigens. Since not every *H. pylori* strain expresses functional BabA or SabA adhesins, other bacterial proteins are most probably also involved in this adhesion process, including LabA (LacdiNAc-binding adhesin), which binds to the LacdiNAc motif on MUC5AC mucin. Besides *H. pylori,* several other gastric non-*Helicobacter pylori Helicobacter*s (NHPH), mainly associated with pigs (*H. suis*) and pets (*H. felis*, *H. bizzozeronii, H. salomonis,* and *H. heilmannii*)*,* may also colonize the human stomach and cause gastric disease, including gastritis, peptic ulcers and mucosa-associated lymphoid tissue (MALT) lymphoma. These NHPH lack homologous to the major known adhesins involved in colonization of the human stomach. In humans, NHPH infection rate is much lower than in the natural hosts. Differences in the glycosylation profile between gastric human and animal mucins acting as glycan receptors for NHPH-associated adhesins, may be involved. The identification and characterization of the key molecules involved in the adhesion of gastric *Helicobacter* species to the gastric mucosa is important to understand the colonization and infection strategies displayed by different members of this genus.

## Glycosylation and Glycan Structures

Glycosylation is a post-translational modification and is defined as the covalent attachment of single sugars (saccharides) to other saccharides, proteins or lipids (Pinho and Reis, 2015; Eichler, 2019; Endo, 2019; Mereiter et al., 2019). This enzyme-mediated glycosidic linkage to specific residues on the target molecules follows the secretory pathway, beginning in the endoplasmic reticulum, in the case of *N*-glycosylation, and finishing in the Golgi apparatus, with major effects on function, stability and subcellular localization of the newly biosynthesized glycoconjugate (Moremen et al., 2012; Pinho and Reis, 2015; Eichler, 2019; Schjoldager et al., 2020). Glycosylation is an essential mechanism for the regulation of several biological processes, such as cell-cell and cell-matrix interaction, protein folding, protection from proteases and subcellular targeting (Eichler, 2019; Endo, 2019). Additionally, glycans have been described as crucial for the interaction and adhesion of several microorganisms to human cells (Etzold and Juge, 2014; Formosa-Dague et al., 2018; Thompson et al., 2019).

Protein glycosylation can be classified in families according to the nature of the linkage, being the biosynthesis and further modifications tissue and cell specific, controlled by glycosyltransferases (Pinho and Reis, 2015). The two main protein glycosylation pathways are the *O-* and *N-*glycosylation, that differ on the amino acid used for the attachment of the glycans. The interaction between the reducing terminal of a *N-*Acetylglucosamine (GlcNAc) with the amine group of an asparagine *via* an aspartylglycosylamine linkage constitutes the first step of the *N-*glycans biosynthesis (Magalhaes et al., 2010; Pinho and Reis, 2015; Eichler, 2019; Endo, 2019; Mereiter et al., 2019). The attachment of the reducing terminal of a *N-*Acetylgalactosamine (GalNAc) residue to the hydroxyl group of a serine or threonine initiates the protein *O-*glycosylation pathway (Schjoldager et al., 2020).

The biosynthesis of the abundant form of GalNAc-type *O*-glycosylation is initiated by a group of enzymes named polypeptide *N-*acetylgalactosamine transferases (ppGalNAc-Ts), which includes a family of 20 enzymes that catalyze the addition of a GalNAc residue (Bennett et al., 2012). The addition of a galactose residue in a β1-3-linkage onto GalNAc-*O*-Ser/Thr originates the formation of the core-1 structure. Next, a second neutral sugar (GlcNAc) can be added onto a β1-6 position to the core one structure, by core two synthases, forming the core-2 structure (Pinho and Reis, 2015; Symmes et al., 2018). These structures can be further elongated by the addition of Gal and GlcNAc residues. Additionally, these glycan structures can be terminally decorated with the addition of sialic acid and fucose residues, by sialyltransferases and fucosyltransferases (Pinho and Reis, 2015; Mereiter et al., 2019). This type of glycosylation is often called mucin type *O*-glycosylation because it is highly abundant in mucins. In addition to the *O-*GalNAc forms, *O-*glycosylation can also occur with fucose (Fuc), mannose (Man), GlcNAc or glucose (Glc) residues (Eichler, 2019).

Mucins are high molecular weight glycoproteins, encoded by over 21 different genes, that can be expressed in a broad range of epithelial tissues, in a tissue-specific manner (Hollingsworth and Swanson, 2004; Duarte et al., 2016). Mucins can be divided into three subgroups: the secreted-gel forming mucins, the cell-surface mucins and the secreted non-gel-forming mucins (Linden et al., 2008). Mucins are important players in the pathogen binding, regulation of antimicrobial responses and induction of tolerance mechanisms (Linden et al., 2008; Magalhaes and Reis, 2010). In fact, mucins are essential for cell protection against pathogens and chemical, enzymatic and mechanical damage, since this mucous layer is the first barrier that these agents need to cross (Linden et al., 2008; Heggelund et al., 2017). The main mucins present on the human healthy gastric mucosa are the membrane-bound MUC1, and the secreted MUC5AC and MUC6, which are the main constituents of the gastric mucous layer (Reis et al., 1997; Reis et al., 2000; Ferreira et al., 2006; Linden et al., 2008; Magalhaes and Reis 2010; Symmes et al., 2018). MUC1 is the most widely studied mucin and is expressed mainly by the foveolar epithelial cells. The secreted MUC5AC is expressed by the foveolar epithelium, while the MUC6 expression is limited to the glands (Reis et al., 1997; Reis et al., 1999; Reis et al., 2000; Linden et al., 2008; Magalhaes and Reis 2010). Occasionally, under pathological conditions, namely intestinal metaplasia, a different pattern of mucins expression can be observed, such as the *de novo* expression of MUC2 (Reis et al., 1999; Teixeira et al., 2002; Linden et al., 2008; Magalhaes and Reis 2010).

Besides these two main types of glycans on proteins, other classes of glycoconjugates are present on the cell, including the proteoglycans and the glycosphingolipids, and other glycosylation forms can also occur on specific types of proteins, namely the Notch receptor (Pinho and Reis, 2015).

## Biosynthesis of Histo-Blood Group Antigens

**FIGURE 1:**
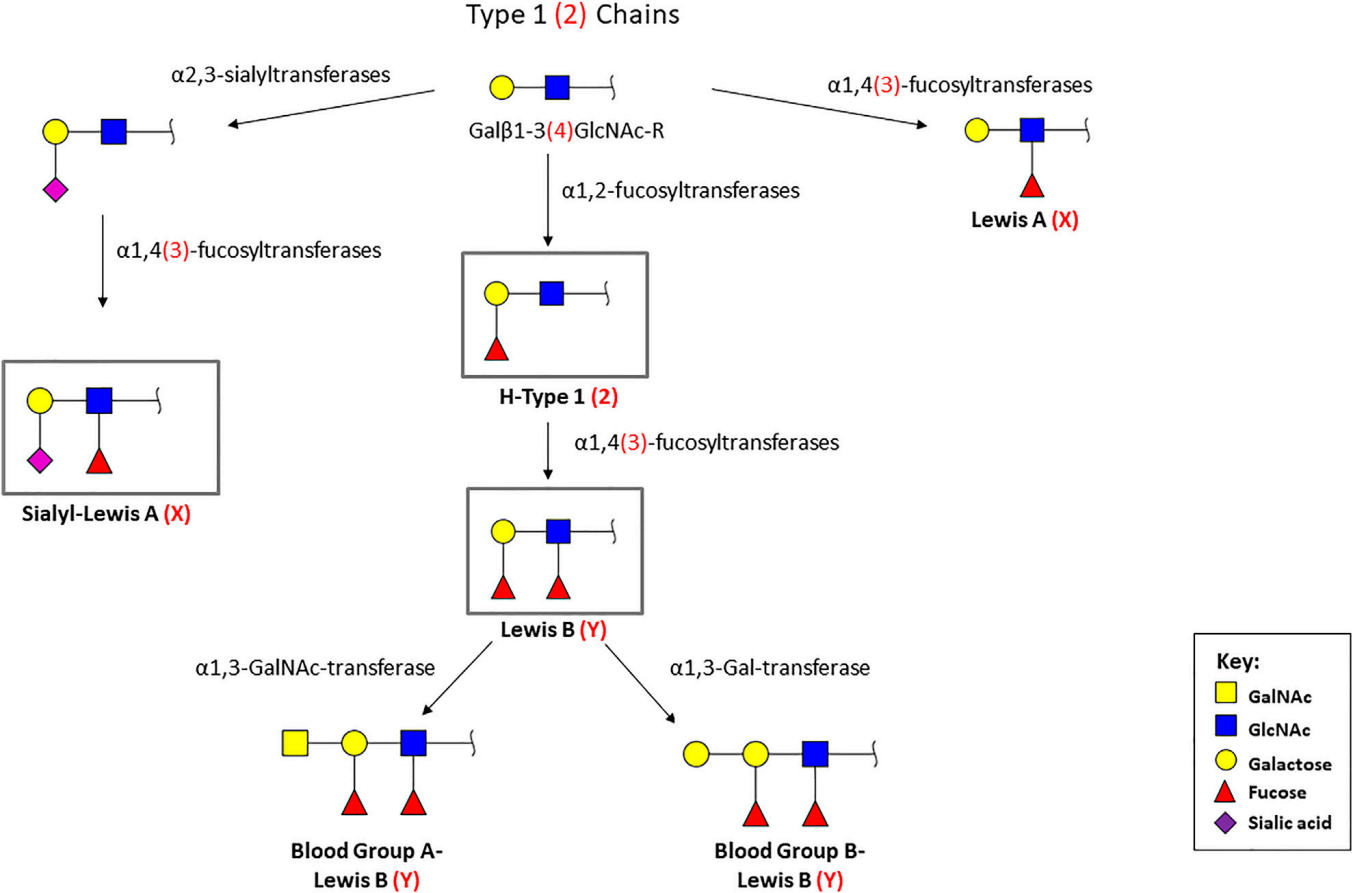
Biosynthetic pathway of the terminal histo-blood group antigens [ABO(H)]. Type 1 chains are characterized by the Galβ1, 3GlcNAc linkage (represented in black), while type 2 chains display a Galβ1, 4GlcNAc linkage (represented between parentheses and in red). Monosaccharide symbols follow the Symbol Nomenclature for Glycans (Neelamegham et al., 2019). The antigens signed in the grey box (LeB, H-type 1, sLeA, and sLeX) are the structures recognized by *H. pylori* BabA and SabA adhesins.

Glycosylation is a highly regulated process important for homeostasis, but alterations have been described to occur in pathological scenarios. The histo-blood group ABO (H) antigens are found in several extracellular fluids, such as saliva and tears, and at the surface of epithelial cells. They mediate the contact with multiple pathogens (De Oliveira et al., 2017; Le Pendu and Ruvoen-Clouet, 2020). The synthesis of the ABO (H) antigens occur by the addition of saccharide residues to both glycoproteins and glycolipids (Yamamoto et al., 1990) (Figure 1). The H phenotype results from the linkage of a fucose to the terminal galactose of the core chains, by an α1,2-fucosyltransferase, resulting in the H antigens 1 and 2 (H-Type 1 and H-Type 2 (Le Pendu et al., 1982; Oriol et al., 1986; Clausen and Hakomori, 1989; Koda et al., 1996; Torrado et al., 2000). The blood-group A and B phenotypes result from the addition of terminal galactose derivates to the H antigens (GalNAcα1, 3 or Galα1, 3), by the action of a GalNAc or a D-galactosyltransferase, respectively (Le Pendu et al., 1982; Oriol et al., 1986; Clausen and Hakomori, 1989; Koda et al., 1996). The Lewis antigens are terminal fucosylated glycan structures that decorate a huge number of glycan chains, which can be classified as type 1 or type 2 Lewis antigens, based on their biosynthesis (Amorim et al., 2014; Magalhaes et al., 2015). The type 1 structures (Lewis A, Lewis B and sialyl-Lewis A) are characterized by the Galβ1,3GlcNAc linkage, while type 2 antigens (Lewis X, Lewis Y and sialyl-Lewis X) display a Galβ1,4GlcNAc linkage (Le Pendu et al., 1982; Oriol et al., 1986; Pinho and Reis, 2015; Symmes et al., 2018). The Lewis A (LeA) and Lewis X (LeX) antigens are formed by the addition of a branched fucose residue to the type 1/2chains (Watkins, 1980; Le Pendu et al., 1982; Oriol et al., 1986; Clausen and Hakomori, 1989; Torrado et al., 2000). On the other hand, Lewis B (LeB) and Lewis Y (LeY) are difucosylated structures, resulting from the attachment of a fucose to the monofucosylated H-Type 1 and H-Type 2 antigens, respectively (Watkins, 1980; Le Pendu et al., 1982; Oriol et al., 1986; Clausen and Hakomori, 1989). The synthesis of these antigens is mediated by several glycosyltransferases, including fucosyltransferases and sialyltransferases, among others (Harduin-Lepers et al., 2012; Pinho and Reis, 2015; De Oliveira et al., 2017).

The H phenotype is the most frequent worldwide (70%), particularly in Western Europe and North America (De Oliveira et al., 2017), and the expression of the ABO (H) blood-group antigens is dependent on the expression of the *FUT2* gene, encoding the fucosyltransferase 2 (Heneghan et al., 1998; De Oliveira et al., 2017). Individuals that express the *FUT2* gene are called secretors, due to the secretion of the corresponding blood-group antigens by mucin-secreting cells (Kelly et al., 1995; Serpa et al., 2004; Magalhaes et al., 2009; Silva et al., 2009; Magalhaes et al., 2016; De Oliveira et al., 2017).

For the secretor individuals, the ABO (H) antigens are expressed by epithelial cells present in many types of tissues (Orntoft et al., 1991; Tsukazaki et al., 1991; Murata et al., 1992; Teixeira et al., 2002; Lee et al., 2006). Due to this localization, these antigens can act as ligands to specific pathogens, such as *H. pylori*, one of the most studied human pathogens.

## Role of Glycans in Host-Pathogen Interaction

The role of glycans in the recognition of pathogens has been widely studied for several types of microorganisms. Indeed, the interaction between the pathogen and the glycan receptors at the cell surface is the first step and is crucial for a successful colonization and infection (Kreisman and Cobb, 2012). The majority of pathogens present glycan binding proteins on their surface that will recognize and bind to specific receptors on the host cell membrane or mucous layer. One of the major players are the mucin *O*-glycans, which are essential for bacterial adhesion, in a species-specific manner (Ringot-Destrez et al., 2017). This binding is crucial for survival of the pathogen in the host microenvironment, since it will help to avoid the elimination by clearance mechanisms and to escape from the immune response (Kreisman and Cobb, 2012; Belotserkovsky et al., 2018). Indeed, the majority of the pathogens are able to modulate the expression of their lectins, in order to adapt to alterations in the host glycosylation profile observed for several diseases (Ringot-Destrez et al., 2017). Glycans have been implicated in host-pathogen interactions of several microorganisms, including viruses such as Influenza virus (Bateman et al., 2010; Air, 2014; Cohen et al., 2016; Byrd-Leotis et al., 2017; Mayr et al., 2018), Calicivirus (Bhella et al., 2008), and recently SARS-Cov-2 (Casalino et al., 2020; Clausen et al., 2020; Watanabe et al., 2020), as well as bacteria, such as *Mycobacterium tuberculosis* (Barnes et al., 2017; Shukla et al., 2018; Fonseca et al., 2020; Matos et al., 2020), *Pseudomonas eruginosa* (Linden et al., 2008), *Escherichia coli* (Krishnan et al., 2014; Bottomley et al., 2020)*, Yersinia pestis, Yersinia pseudotuberculosis* and *Yersinia enterocolitica* (Pakharukova et al., 2016) and *H. pylori* (Borén et al., 1994; Aspholm-Hurtig et al., 2004; Benktander et al., 2012).

## *Helicobacter pylori* Adhesion to Human Gastric Mucosa: An Interaction Mediated by Glycans

*Helicobacter pylori* is a Gram-negative, microaerophilic, spiral-shaped bacterium, that colonizes the stomach of half of the worldwide human population (Altman et al., 2005; Magalhaes and Reis, 2010; Huang et al., 2016; Ansari and Yamaoka, 2017). A persistent infection with *H. pylori* results in chronic gastritis, which can evolve to peptic ulcers, precancerous lesions (atrophy, intestinal metaplasia and dysplasia) and gastric adenocarcinoma (Huang et al., 2016; Ansari and Yamaoka, 2017). Indeed, *H. pylori* is considered a group I carcinogen due to its strong association with gastric carcinoma, with a 2-8-fold increased risk vs. the non-infected population (Huang et al., 2016; Ansari and Yamaoka, 2017). Fortunately, in most cases, *H. pylori* infection remains asymptomatic, however 10% of the individuals develop severe gastric lesions and of these, 1–3% progresses to gastric carcinoma with a low 5-years survival rate (De Falco et al., 2015; Huang et al., 2016). Furthermore, *H. pylori* may also play a role in non-gastric diseases, such as Parkinson’s disease, Alzheimer’s disease, diabetes mellitus, hematologic disorders and asthma, which might be associated with changes in host immune response and human microbiome induced by an infection with this bacterium (Bravo et al., 2018; Menard and Smet, 2019; Gottesmann et al., 2020).

Infection with *H. pylori* is usually clustered in families, and individuals are normally infected during childhood and, if not eradicated, it can persist through individual’s lifetime (Clyne and May, 2019). The prevalence of this pathogen varies within the geographic region and depends on several factors, such as age, socio-economic status, educational level and hygiene (De Falco et al., 2015; Huang et al., 2016). The transmission mechanism is not completely known but it may occur *via* fecal-oral or oral-oral route (Huang et al., 2016; Ansari and Yamaoka, 2017).

**FIGURE 2:**
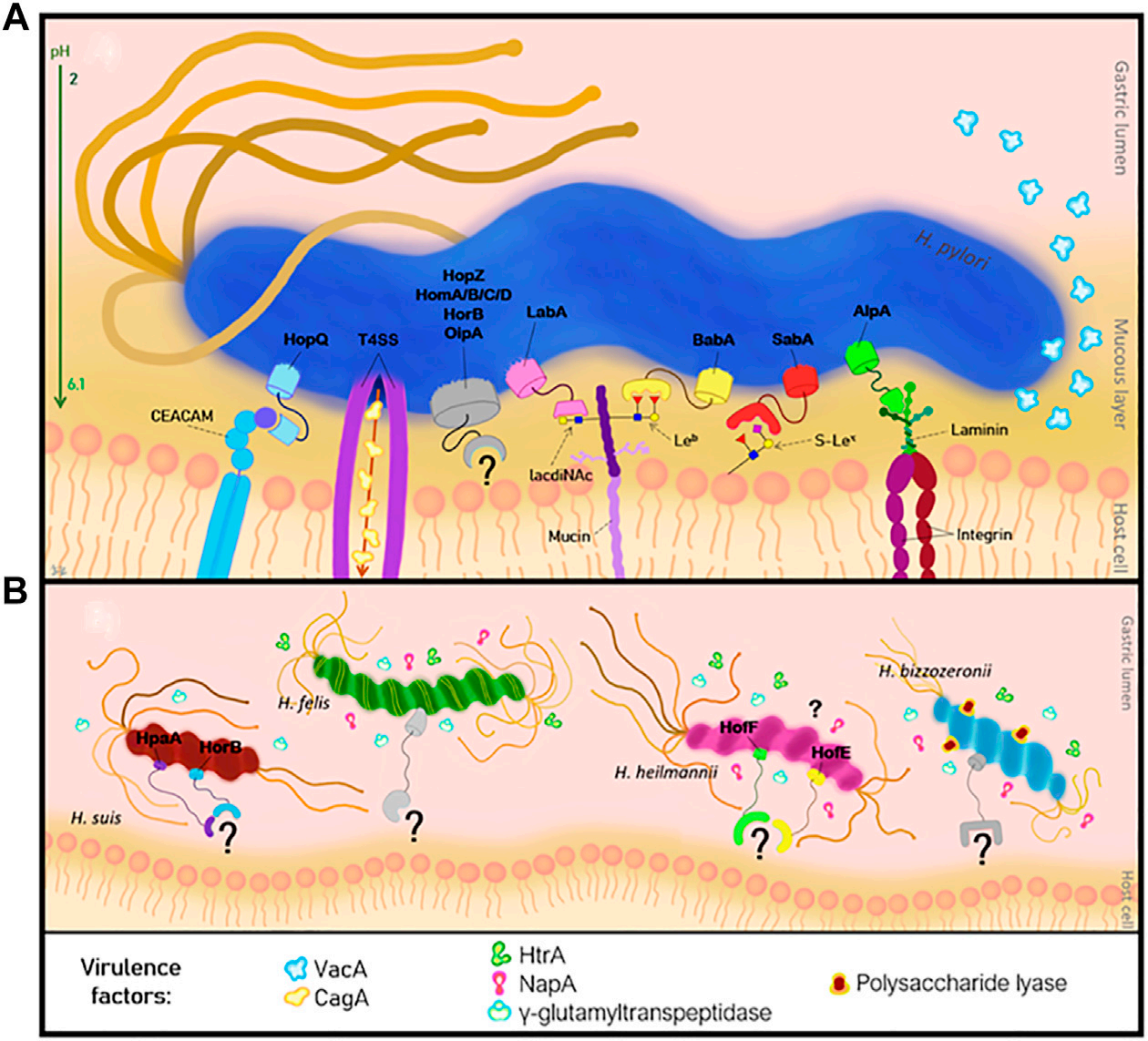
Pathogenesis of *Helicobacter* infection of the human gastric mucosa. **(A)** Interaction of *H. pylori* adhesins with the host cell receptors, and the virulence factors. **(B)** NHPH adhesins and virulence factors involved in the adhesion mechanism.

*H. pylori* uses several virulence factors to colonize the human gastric mucosa (Ansari and Yamaoka, 2017). The virulence factors expressed by *H. pylori* include the CagA (cytotoxin-associated gene A) and the VacA (vacuolating cytotoxin A) molecules (Marcos et al., 2008; Ansari and Yamaoka, 2017). Besides these factors, the bacterial genome encodes a large number of outer membrane proteins (OMP), which are expressed at the bacterial cell envelop and can be divided into six families: the *H. pylori* OMP, the Hop-related, the *Helicobacter* OMP, the *Helicobacter* outer membrane (Hom), the iron-regulated OMPs and the efflux pump OMPs (Magalhaes and Reis, 2010; Ansari and Yamaoka, 2017; Bauwens et al., 2018). The majority of the OMPs belong to the Hop proteins, and can act as transport channels (porins) or adhesins such as the blood-group antigen binding adhesin (BabA) (Borén et al., 1993), the sialic acid-binding adhesin (SabA) (Borén et al., 1994), the lacdiNAc-binding adhesin (LabA) (Rossez et al., 2014), the adherence-associated lipoprotein A/B (AlpA/B) and the outer inflammatory protein A (OipA), the HopQ and the HopZ (Benktander et al., 2012; Ansari and Yamaoka, 2017; Bauwens et al., 2018; Benktander et al., 2018). The attachment of the adhesins present on the bacterial surface to the gastric mucus layer is the initial step and is crucial for the colonization and subsequent infection by *H. pylori* (Rossez et al., 2014; Huang et al., 2016; Ansari and Yamaoka, 2017). The binding to the gastric epithelium is essential for a successful colonization because it will protect the bacteria from clearance mechanisms, such as liquid flow, peristaltic movement, washing and mucus shedding (Aspholm et al., 2006; Huang et al., 2016; Ansari and Yamaoka, 2017). Additionally, attachment provides a source of nutrients to the bacteria and promotes the delivery of bacterial toxins or other virulence factors to the host cells, contributing to the development of a persistent infection (Aspholm et al., 2006; Ansari and Yamaoka, 2017) (Figure 2).

*H. pylori* can be classified based on its ability to bind to the blood-group antigens: specialist strains bind to blood-group O antigens, whereas the generalist strains bind to group O, A and B antigens (Ansari and Yamaoka, 2017). Besides this, the triple positive strains (BabA-, VacA- and CagA-positive) are associated with more severe gastric diseases, namely peptic ulcers and gastric carcinoma (Benktander et al., 2012; Benktander et al., 2018).

A successful infection occurs in two steps: 1) the bacteria reach the surface of the gastric mucosa, which has a nearly neutral pH, after crossing the mucus layer; and 2) the bacteria adhere to the gastric epithelial cells via the OMPs (Borén et al., 1994; Borén et al., 1993; Huang et al., 2016). The first interaction is mediated by the BabA adhesin, which recognizes and binds to fucosylated blood-group antigens H-type 1 and LeB (Borén et al., 1993; Ilver et al., 1998; McGuckin et al., 2011; Benktander et al., 2012). The LeB antigen, is the main receptor for the BabA adhesin, and it can be expressed on MUC5AC mucin, on MUC1 and MUC2 mucins (Ansari and Yamaoka, 2017; Mahdavi et al., 2013) (Table 1). The MUC5AC mucin is co-expressed with the LeB and LeA antigens by the gastric epithelial cells (Linden et al., 2008; Magalhaes et al., 2010; Ansari and Yamaoka, 2017). On the other hand, MUC6 mucin is co-expressed with the type 2 Lewis antigens (LeX and LeY) (Reis et al., 2000).

**TABLE 1:** *Helicobacter pylori* adhesins and their described glycan ligands.

Adhesin	Receptors identified	Localization	Reference
BabA	Lewis b blood group antigen (LeB) and terminal fucose, H1-antigen, A-antigen and B-antigen	Gastric epithelium	Borén et al. (1993); Ilver et al. (1998); Benktander et al. (2012); McGuckin et al. (2011)
SabA	sialyl-Lewis A blood group antigen (sLeA)	Gastric epithelium	Madhavi et al. (2002)
	sialyl-Lewis X blood group antigen (sLeX)	Gastric epithelium	Madhavi et al. (2002)
LabA	lacdiNAc motif	Gastric epithelium	Rossez et al. (2014); Benktander et al. (2018)

The binding mechanism of the *H. pylori* BabA adhesin to the carbohydrate receptors, more specifically to LeB, has been studied in detail. Recently, Moonens *et al.,* revealed the X-ray structures of representative specialist and generalist BabA isoforms (Moonens et al., 2016). This analyses demonstrated that the preference shift results from a single amino acid substitution in the carbohydrate binding domain, which is constituted by one conserved loop (CL2) and two diversity loops (DL1 and DL2) (Moonens et al., 2016). In addition, it was showed that the DL1 is responsible for the specialist or generalist BabA binding: the specialist binds to the blood group O trough the CL2 and DL2 loops, while the binding of generalists involves the CL2 and both DL1 and DL2 (Moonens et al., 2016). Furthermore, the biophysical properties and the strength of the binding of the *H. pylori* BabA adhesin to the LeB antigen was evaluated by atomic force microscopy (Parreira et al., 2014). Detailed analysis showed the presence of two different bond populations, suggesting that the binding follows a two-state model, to improve the adhesion efficiency and stability (Parreira et al., 2014). Additionally, Hage *et al.*, also evaluated the molecular aspects of the BabA-LeB interaction, showing that the binding involves two fucose residues (Fuc1 and Fuc4), two galactose residues (Gal2 and Gal5), one *N*-Acetylglucosamine residue (GlcNAc3) and one glucose residue (Glc6) (Hage N 2015). Furthermore, the amino acids in the BabA adhesin involved in the binding to each LeB glycan residue were also identified (Hage et al., 2015).

*H. pylori* is very sensitive to the low pH of the gastric fluid, and the bacteria use unique strategies to survive in this harsh microenvironment (Ansari and Yamaoka, 2017; Gottesmann et al., 2020). The helical shape and the flagella, along with the urease production, favors *H. pylori* entrance and penetration within the gastric mucus, following a pH gradient (Gottesmann et al., 2020)*.* Recently, it has been described that the binding of the BabA adhesin to its ligands is also pH responsive (Bugaytsova et al., 2017). Given the acidic gastric environment, the adherence of *H. pylori* and its strength linkage to the ABO glycan antigens have been shown to be crucial for the survival in this unique environment (Bugaytsova et al., 2017). Under low pH, the linkage is suppressed, and the bacteria are released and relocated into a more neutral microenvironment, escaping to physiological events such as the shedding of epithelial cells and mucus turnover (Bugaytsova et al., 2017).

The non-secretor phenotype is characterized by the absence of ABO (H) antigens on gastric epithelial cells, which express only simple fucosylated Lewis structures (Heggelund et al., 2017). In these individuals, the colonization by *H. pylori* is mediated by the interaction between SabA and the sialylated glycans (Benktander et al., 2018), suggesting that the BabA-LeB interaction is important but not essential for colonization. However, it has been demonstrated that infection with *H. pylori* BabA-positive strains, as well the secretor status of the individual are associated with increased risk for development of peptic ulcer disease in Western countries, where the secretor phenotype prevails (Ansari and Yamaoka, 2017).

The majority of the bacteria are located in the gastric pits and in the mucus, and only a small amount colonizes deeper portions and are attached to gastric cells (Clyne and May, 2019). The specific localization of *H. pylori* has been widely investigated and some hypotheses have been formulated, based on the glycans expressed on this region. The deeper glands are characterized by the expression of MUC6 and α1, 4-GlcNAc residues, which are attached to core 2-branched *O-*glycans (Kawakubo et al., 2004; Ferreira et al., 2006). *In vitro* studies have demonstrated that the biosynthesis of a major *H. pylori* cell wall component can be inhibited by these α1, 4-GlcNAc-capped *O-*glycans, which can preclude bacterial growth, and function as a natural antibiotic (Kawakubo et al., 2004).

Colonization of the human gastric mucosa by *H. pylori* results in inflammation, which is accompanied by a remodeling of the glycosylation profile, with *de novo* expression of α2, 3-sialylated glycans, namely sialyl-Lewis A (sLeA) and sialyl-Lewis X (sLeX) (Mahdavi et al., 2002). These sialylated structures are recognized by SabA adhesin, which constitutes a second point of attachment to the host gastric mucosa and is important for the colonization process (Mahdavi et al., 2002; Linden et al., 2008; Magalhaes et al., 2015; Benktander et al., 2018) (Table 1).

An additional adhesin was suggested to play a role in *H. pylori* interaction with the human gastric mucosa: the lacdiNAc-binding adhesin (LabA) (Rossez et al., 2014; Benktander et al., 2018). This adhesin was first described as binding specifically to the lacdiNAc motif on MUC5AC mucins (Rossez et al., 2014). Recently, structural analysis of LabA suggested that the lacdiNAc motif might not be the ligand for this adhesin (Mthembu et al., 2020; Paraskevopoulou et al., 2021). Thus, the role of LabA and the binding specificity to lacdiNAc residues remains unclear and further studies are needed to elucidate this interaction (Mthembu et al., 2020; Paraskevopoulou et al., 2021).

The efficacy of the conventional *H. pylori* eradication therapy, usually a combination of 2-3 antibiotics with an acid-suppressive drug (Chey et al., 2017), has been declining in the last years, and the WHO considered priority the search for new drugs and alternative therapies (De et al., 2009; Blaecher et al., 2013). One of the major problems has been the acquisition of antimicrobial resistance, not only for *H. pylori*, but also for some NHPH such as *H. heilmannii, H. ailurogastricus, H. felis* and *H. suis* (Van den Bulck et al., 2005; Berlamont et al., 2019; Matos et al., 2020). The analysis of the antimicrobial susceptibility pattern is important to better understand the factors underneath the increasing resistance and to improve the available treatment.

On the other hand, since the search of new anti-adhesion drugs is urgent, the BabA mediated adhesion and the pH-responsive detachment and reattachment has been the aim of several anti-*H. pylori* adhesion studies. The first investigation were based on the anti-adhesive properties of the 3′-sialyllactose (Mysore et al., 1999; Parente et al., 2003), but this molecule was unable to prevent bacterial colonization. Additionally, several other molecules have been evaluated, including polyphenols (Shmuely et al., 2004; Hensel et al., 2007; Wittschier et al., 2007; Niehues et al., 2010), peptides (Niehues et al., 2010) and polysaccharides (Lengsfeld et al., 2007; Wittschier et al., 2009; Messing et al., 2014). The anti-adhesive properties of these molecules have been widely demonstrated however, it is not clear which bacterial OMPs are being targeted by each compound and which interactions are being blocked (Gottesmann et al., 2020). Besides these, another interesting approach is the use of glycans and their characteristics to produce antiadhesion microspheres, namely chitosan-microspheres (Gonçalves et al., 2013; Henriques et al., 2020) and glycan-coated microspheres (Goncalves et al., 2016) to impair *H. pylori* colonization*.*

## Alternative Players in *Helicobacter pylori* Adhesion

The lipopolysaccharide (LPS) is a structural component present in the outer cell wall of Gram-negative bacteria and may serve as a docking to the host cell (Gottesmann et al., 2020). The toxicity of the LPS from *H. pylori* is low compared to that of several other Gram-negative bacteria (Monteiro et al., 1998). One of the major components of the LPS is the O antigen composed of repeating units of sugars (Van Amersfoort et al., 2003). Biochemical analysis demonstrated that O antigen of *H. pylori* is homologous to the Lewis blood group antigens H-type 1, LeA, LeX and LeY (Monteiro et al., 1998; Altman et al., 2005). The expression of these structures in the bacterial surface mimics the glycans expressed by the gastric epithelial cells, which might be helpful in colonization (Altman et al., 2005). Furthermore, it has been suggested that the *H. pylori* O-antigen sidechains can induce the expression of LeX antigen, which may promote the adhesion of the bacteria to the gastric epithelial cells (Edwards et al., 2000; Mahdavi et al., 2003). However, the exact role of this antigen in the colonization process remains unknown (Mahdavi et al., 2003).

*H. pylori* also possess a type IV secretion system, which is helpful for the adhesion and fixation on the host cell surface (Chmiela and Kupcinskas 2019). The BabA-LeB interaction can also help in the bacterial fixation to the cell membrane, which will allow the transfer of bacterial virulence factors into the host cells (Gottesmann et al., 2020).

The *H. pylori* adhesion to the human gastric mucosa trough BabA and SabA adhesins is well-characterized (Jin et al., 2018). Detailed analysis of the glycoconjugates present in the human stomach, from individuals with different blood groups, showed a high degree of structural complexity. Furthermore, the composition of glycosphingolipids composition seems to be different according to the blood group, being the type 2 chains the dominant core chain (Jin et al., 2018). Interestingly, it was demonstrated that *H. pylori* did not bind to the O (Rh-) P blood group, whose major complex glycosphingolipids are the LeX, LeA and H-type 2 pentaosylceramides and LeY hexaosylceramides (Jin et al., 2018). In the other blood groups, glycosphingolipids present on the stomach of these individuals are able to bind to several *H. pylori* compounds (Jin et al., 2018).

## Non-*Helicobacter Pylori Helicobacter*s (NHPH) Adhesion: What Is the Mechanism and Which Molecules Are Involved?

*H. pylori* is the most prevalent *Helicobacter* species in the human stomach, however other gastric, spiral-shaped NHPH have also been associated with the development of gastric disorders in humans (Baele et al., 2008; Ali et al., 2018; Matos et al., 2020). NHPH have been detected in 0.2–6% of the human gastric biopsies, usually accompanied by gastritis, antral erosions, duodenal ulcers and gastric MALT lymphoma (Morgner et al., 2000; Haesebrouck et al., 2009; Joosten et al., 2016; Ali et al., 2018). So far, H. suis, H. felis, H. bizzozeronii, H. salomonis, and H. heilmannii are the NHPH species detected in the human stomach (Boyanova et al., 2003; Sýkora et al., 2003; Haesebrouck et al., 2009; Yakoob et al., 2012). *H. suis* is mainly associated with pigs and non-human primates, whereas the other gastric NHPH preferentially colonize the canine and feline stomach. However, occasionally they can cross the host species barrier and infect humans (Haesebrouck et al., 2009; Amorim et al., 2014; Bento-Miranda and Figueiredo, 2014; Liu et al., 2015). Compared to the natural hosts, NHPH colonization of the human stomach is lower and more focal (Haesebrouck et al., 2009).

Detailed analysis demonstrated that NHPH species lack major *H. pylori* adhesins described so far, such as BabA, SabA or AlpA/B (De Witte et al., 2016; Padra et al., 2018). The genome of *H. bizzozeronii*, *H. felis*, *H. salomonis* and *H. heilmannii* encodes several OMPs but lacks genes encoding the Bab and Sab adhesins (Arnold et al., 2011; Schott et al*.* 2011a; Smet et al., 2013). Furthermore, the alpA and alpB genes are not present in *H. felis*, *H. heilmannii* and *H. bizzozeronnii* (Odenbreit et al., 1999; Schott et al., 2011b). Even without the main molecules used by *H. pylori* for colonization, the NHPH species are still able to colonize the stomach from both humans and animals. So the question is: which are the adhesion molecules used by NHPH? The answer is not clear, and the full mechanisms and the intervenients remain to be elucidated.

Biochemical studies on *H. heilmannii* showed the expression of the OMPs HofE and HofF, which were identified as adhesins (Cheng et al., 2016) (Figure 2). These OMPS are involved in binding of this bacterium to the gastric mucosa. They present higher affinity to gastric epithelial cells than mucins, and they are essential for the activation of IL-1β which on its turn induces MUC13 expression on the gastric epithelium (Cheng et al., 2016; De Witte et al., 2016). *H. suis* is the most prevalent NHPH found in the human stomach (Haesebrouck et al., 2009; Padra et al., 2018) and a remarkable high prevalence of *H. suis* DNA (i.e. 27%) was detected in gastric biopsies from human patients with idiopathic parkinsonism (Blaecher et al., 2013). Like the other gastric NHPH, *H. suis* does not express major adhesins involved in colonization of the human stomach by *H. pylori*. However, it expresses some *H. pylori* similar OMPs, namely HorB and HpaA (*H. pylori* adhesin A) (Padra et al., 2018) (Figure 2). *In vitro* studies demonstrated that the porcine stomach expresses mucins at the surface epithelium, that resemble to human MUC5AC and MUC6 mucins and may constitute a binding site for *H. suis* (Padra et al., 2018). However, the identity of these mucins, the glycans and the specific *H. suis* adhesins are not known yet (Padra et al., 2018). Structural analysis of the porcine gastric mucins showed that these are large, oligomeric and highly glycosylated structures, similar to the human mucins (Aspholm et al., 2006). The major difference was at mucins sulfation level, that is very frequent among porcine mucins, but virtually absent in mucins of a healthy human stomach (Padra et al., 2018). This difference might help to explain the different colonization pattern of *H. suis* in the human and porcine stomach. Compared to the porcine stomach, colonization of the human stomach is indeed more limited and much more focal (Haesebrouck et al., 2009). Similar to the human stomach, a pH gradient across the mucus layer is observed in the porcine stomach, ranging from acidic in the lumen to neutral at the epithelial surface (Padra et al., 2018). Additionally, *in vitro* studies demonstrated that *H. suis* is able to bind to Galβ3GlcNAcβ4Glc glycoconjugates at neutral and acidic pH and binds to negatively charged glycans at acidic pH (Padra et al., 2018). Furthermore, it was observed that Galβ3GlcNAcβ3Galβ4Glc structures can inhibit the binding of *H. suis* to mucins (Padra et al., 2018).

*H. suis* and *H. heilmannii* are two NHPH species that have been widely studied and recently their *in vitro* adhesion ability to human gastric cell lines was evaluated (Berlamont et al., 2020). Interestingly, both *Helicobacter* species were able to bind to the gastric epithelial cells *in vitro,* but detailed analysis showed that frequently two genes were up-regulated and four genes were down-regulated in both pathogens (Berlamont et al., 2020). BLASTp analysis demonstrated that the genes differentially expressed by *H. suis* and *H. heilmannii* belong to several functional classes, which suggests that the *in vitro* adhesion of both strains to human gastric cells demands pleiotropic adaptive responses (Berlamont et al., 2020). However, the clinical significance of these findings and the complete pathway involved in the adhesion process needs to be better elucidated (Berlamont et al., 2020).

The canine gastric mucosa is one of the preferential niches for *H. felis*, *H. bizzozeronii*, *H. heilmannii* and, less often, *H. salomonis* (Amorim et al., 2014). Analysis of the canine glycophenotype showed that the canine gastric mucosa is characterized by absence of LeB and sLeA antigens expression and a slight expression of LeA (Amorim et al., 2014). The minor expression of type 1 Lewis antigens could be explained by the lack of α1, 4-fucosyltransferase activity in canine gastric epithelial cells (Kulhlmann et al., 1983; Amorim et al., 2014). On the other hand, type 2 Lewis antigens, mostly LeX and LeY are strongly expressed at the canine gastric superficial and foveolar epithelium (Amorim et al., 2014). Furthermore, it was demonstrated that the dog orthologous of human *FUT3* and *FUT5* genes present an arginine residue, which is compatible to the α1, 3-fucosyltransferase activity, instead of the α1, 4-fucosyltransferase observed in humans, further resulting in type 2 Lewis antigens biosynthesis by the dog gastric epithelial cells (Kulhlmann et al., 1983; Amorim et al., 2014). The different glycophenotype, together with the absence of BabA homologs, could explain the low infection rate by canine NHPH in humans, and concomitantly, the very rare colonization of dogs with *H. pylori*.

Both *H. pylori* and NHPH share virulence factors involved in epithelial cell death such as the ɣ-glutamyl transpeptidase (Haesebrouck et al., 2009). In addition, genes encoding homologues of other *H. pylori* virulence factors such as the secreted serine protease HtrA and the immunomodulator NapA are present in *H. felis*, *H. bizzozeronnii* and *H. heilmannii s.s*. genomes. In *H. bizzozeronnii* a polysaccharide lyase was regarded as a potential new virulence factor (Schott et al., 2011a). Later Kondadi *et al.* (Kondadi et al., 2012), characterized a novel lipopolysaccharide α2, 3-sialyltransferase from *H. bizzozeronii* that showed a preference for N-acetyllactosamine as a substrate. The authors showed that the expression of a terminal 3′sialyl-LacNAc on LPS is a phase-variable characteristic of both human- and canine derived *H. bizzozeronii* strains. Moreover, the authors suggest that this sialylated structure can mimic the surface glycans of the host mammalian cells (Kondadi et al., 2012).

## Final Remarks

*H. pylori* infects half of the worldwide population, being associated with several gastric diseases. The interaction between *H. pylori* and the human gastric mucosa has been well characterized, and several intervenients have been described. The adhesion of *H. pylori* through the interaction of the BabA adhesin to both LeB and H-type 1 glycans, expressed by the gastric epithelial cells of healthy secretors, results in the initiation of an inflammatory response, with *de novo* synthesis of sialylated structures, such as sLeA and sLeX. These are carbohydrate antigens that are recognized by the SabA adhesin expressed at the bacterial surface. Gastric NHPH that are primary associated with pigs, dogs, and cats, may also colonize the human stomach resulting in gastric disorders. However, the precise adhesion mechanisms of NHPH, including the adhesins and ligands responsible for the colonization, are almost unknown. Further studies, including the screening of NHPH OMPs using glycan arrays will be very helpful in the identification of the players in adhesion, improving and contributing to a thorough knowledge about human NHPH infection.
